
JOURNAL INFORMATION
==============================
NLM Title Abbreviation: ScientificWorldJournal
Journal ID: ScientificWorldJournal
NLM Journal ID: 101131163
Journal ID: TSWJ

The Scientific World Journal

ISSN: 2356-6140
EISSN: 1537-744X
Publisher: Wiley

ARTICLE INFORMATION
==============================
PMCID: PMC6009258
PMID: 29973779
DOI: 10.1100/tsw.2002.5
Article ID: 267475
Article version: 1
Subjects: Short Report

Genome Analysis of Pass2 a Semi-Automated Database of Protein Alignments Organised as Structural Superfamilies

Bhaduri Anirban
Mallika V.
Sowdhamini R.
National Centre For Biological Sciences, UAS-GKVK Campus, Bangalore 560 065, India
Publication date: 2002
Electronic publication date: 2002 May 16
Volume: 2
First page: 9
Last page: 10
Copyright: Copyright © 2002 Anirban Bhaduri et al.
Copyright year: 2002
License: This is an open access article distributed under the Creative Commons Attribution License, which permits unrestricted use, distribution, and reproduction in any medium, provided the original work is properly cited.
License URL: https://creativecommons.org/licenses/by/3.0/

Funding: Wellcome Trust

==============================
Advances in Gene Technology: The Genome and Beyond –
Structural Biology for Medicine (Proceedings of the 2002 Miami
*Nature Biotechnology* Winter Symposium)
*TheScientificWorld* 2002, 2(S2), 9–10
ISSN 1532-2246; DOI 10.1100/tsw.2002.5

### GENOME ANALYSIS OF PASS2 A SEMI-AUTOMATED DATABASE OF PROTEIN ALIGNMENTS ORGANISED AS STRUCTURAL SUPERFAMILIES

Anirban Bhaduri,V. Mallika, and R. Sowdhamini
National Centre For Biological Sciences, UAS-GKVK Campus, Bangalore 560 065

**INTRODUCTION**. Success of genomic sequencing projects has lead to an ever-increasing gap in the number of protein sequences and structure that are deposited in the databanks[1,2]. Homologous sequences sharing a degree of sequence, structural, and functional similarity[3,4] can be grouped though simple search programs. According to SCOP[5] (a dictionary of protein structural entrie), Superfamily of proteins are those sharing an insignificant sequence similarity yet possessing a similar function and fold, maintaining an evolutionary relationship among themselves. CAMPASS[6] forms the first version of a protein superfamily database, corresponding to 69 superfamilies, which records the alignments of proteins aligned using COMPARER[7]. Availability of such alignment databases over the World Wide Web offers the possibility to study and design experiments on specific superfamilies; they also permit systematic survey and analysis of various structural properties and to perform fold predictions. Analyses of structural and sequence differences amongst known superfamily members can hopefully provide useful guidelines for modelling distant related proteins. We report the genome analysis conducted for each Superfamily member for the semi-automated updated version of the superfamily alignment database, which has been designed to be in direct correspondence with SCOP database (1.53 release; [5]). The updated version of the database (PASS2) has 110 multimember superfamilies and 613 single member superfamilies. The analysis was conducted across 72 genomes (41 complete).

**METHODS.**

**Selection of the Members for the Database.** One representative protein domain entry, of the best resolution and R-factor from each family, is chosen for a preliminary alignment. NMR structures are considered equivalent to a 3.2A resolution crystal structure in the present context. Protein structural coordinates where been obtained from Protein Data Bank[2] and protein domain coordinates of the desired chain and domain boundaries considered in the superfamily are extracted using CHAINRESALL (unpublished) program. ATM2SEQ[8] is used to obtain the corresponding amino acid sequences and MALIGN[8] for a multiple-sequence alignment using a constant gap penalty of 40. MOTIFS (unpublished) provides a percentage identity matrix which is examined, to derive a nonredundant representative set of protein domains for the superfamily such that, no two proteins are more than 27% identical by the MALIGN alignment.

**Genome Analysis of the Members of the Database**. PSI-BLAST[9], a sensitive method for identifying distant relatives and convenient for automatic searches was employed for genome analysis. The search was carried across 72 genomes out of which 41 were complete. Such searches were performed with 10 iterations and an E-value of 0.01, using each of the representative Superfamily members as a query against the genome databases. The genome sequences, which are either homologues or additional superfamily members, are aligned with the original structure-based alignment and reannotated using JOY[9]. Where possible, links to such structure-annotated alignments with genome sequence homologues of superfamily members are provided.

**DISCUSSION.** Genome analysis done for the members of PASS2 provides an overview of the nature of distribution of different superfamily in the different genomes. The JOY[9] annotation of the Superfamily member shows the structural environment of individual residues on the sequence alignment. Since the hits in the genomes where aligned along with the JOY annotated representative Superfamily member, one can have a notion of the plausible structural environment in these homologues though the structure may not known. One may access the database at http://www.ncbs.res.in/%7Efaculty/mini/campass/pass.html

**ACKNOWLEDGEMENT.** Dr. R. Sowdhamini is a recipient of Wellcome Trust Senior Research Fellowship and V. Mallika is supported by the Wellcome Trust.

**REFERENCES**

1. Bairoch, A. and Apweiler, R. (2000) Nucl. Acids Res. 28, 45–48.
2. Bernstein, F.C., Koetzle, T.F., Williams, G.J.B., Meyer, E.F., Brice, M.D., Rodgers, J.R., Kennard, O., Shimanouchi, T., and Tasumi, M. (1977) J. Mol. Biol. 112, 535–542.
3. Richardon, J.S. (1981) Adv. Prot. Chem. 34, 167–339.
4. Murzin, A.G., Brenner, S.E., Hubbard, T., and Chothia, C. (1995) J. Mol. Biol. 247, 536–540.
5. Sowdhamini, R., Burke, D.F., Huang, J.F., Mizuguchi, K., Nagarajaram, H.A., Srinivasan, N., Steward, R.E., and Blundell, T.L. (1998) Structure 6, 1087–1094.
6. Sali, A. and Blundell, T.L. (1990) J. Mol. Biol. 212, 403–428.
7. Johnson, M.S., Overington, J.P., and Blundell, T.L. (1993) J. Mol. Biol. 231, 735–752.
8. Altschul, S.F., Madden, T.L., Schaffer, A.A., Zhang, J., Zhang, Z., Miller, W., and Lipman, D.J. (1997) Nucl. Acids Res. 25, 3389–3402.
9. Mizuguchi, K., Deane, C.M., Blundell, T.L., Johnson, M.S., and Overington, J.P. (1998) Bioinformatics 14, 617–623.
